# GRAding of functional and anatomical response to DExamethasone implant in patients with Diabetic Macular Edema: GRADE-DME Study

**DOI:** 10.1038/s41598-020-79288-w

**Published:** 2021-02-26

**Authors:** Patricio J. Rodríguez-Valdés, Matus Rehak, Dinah Zur, Anna Sala-Puigdollers, Samantha Fraser-Bell, Marco Lupidi, Jay Chhablani, Zafer Cebeci, Inês Laíns, Voraporn Chaikitmongkol, Adrian T. Fung, Mali Okada, Jan Darius Unterlauft, Lital Smadar, Anat Loewenstein, Matias Iglicki, Catharina Busch

**Affiliations:** 1 Instituto de Oftalmología y Ciencias Visuales, Escuela de Medicina, Tecnológico de Monterrey, Monterrey, Mexico; 2 Department of Ophthalmology, University Hospital Leipzig, Liebigstr. 10-14, 04103 Leipzig, Germany; 3 Division of Ophthalmology, Tel Aviv Sourasky Medical Center, Tel Aviv, Israel; 4 Sackler Faculty of Medicine, Tel Aviv University, Tel Aviv, Israel; 5 Institut Clínic d’Oftalmología (ICOF), Hospital Clinic de Barcelona, Barcelona, Spain; 6 Department of Ophthalmology, Sydney University, Sydney, Australia; 7 Department of Surgical and Biomedical Sciences, Section of Ophthalmology, University of Perugia, Perugia, Italy; 8 UPMC Eye Center, University of Pittsburgh, Pittsburgh, USA; 9 L.V. Prasad Eye Institute, Banjara Hills, Hyderabad, India; 10 Department of Ophthalmology, Istanbul Faculty of Medicine, Istanbul University, Istanbul, Turkey; 11 Massachusetts Eye and Ear, Harvard Medical School, Boston, USA; 12 Association for Innovation and Biomedical Research on Light, Coimbra, Portugal; 13 Retina Division, Department of Ophthalmology, Faculty of Medicine, Chiang Mai University, Chiang Mai, Thailand; 14 Westmead and Central Clinical Schools, Discipline of Clinical Ophthalmology and Eye Health, The University of Sydney, Sydney, Australia; 15 Department of Ophthalmology, Faculty of Medicine, Health and Human Sciences, Macquarie University, Sydney, Australia; 16 Royal Victorian Eye and Ear Hospital, Melbourne, VIC Australia; 17 Incumbent, Sydney A. Fox Chair in Ophthalmology, Tel Aviv University, Tel Aviv, Israel; 18 Private Retina Service, University of Buenos Aires, Buenos Aires, Argentina

**Keywords:** Drug therapy, Retinal diseases, Diabetes complications

## Abstract

To analyze functional and anatomical response patterns to dexamethasone (DEX) implant in diabetic macular edema (DME), to describe proportion of responders and non-responders, and to propose a new DME grading system. Retrospective, multicenter, observational cohort study. Naïve and non-naïve DME patients were treated with DEX, with visual acuity (VA) ≥ 0.2 logMAR and central subfield thickness (CST) of ≥ 300 µm. Functional and anatomical responses were graded after 2 and 4 months, and categorized as early and stable improvement, early and progressive improvement, pendular response, delayed improvement, and persistent non-response. 417 eyes were included (175 treatment naïve eyes). Compared to non-naïve eyes, naïve eyes showed a very good functional response (VA gain ≥ 10 letters) more frequently after 2 and 4 months (56% and 57% [naïve] vs. 33% and 28% [non-naïve], p < 0.001). A VA gain < 5 letters (non-response) after 2 and 4 months was seen in 18% and 16% of naïve eyes, and in 49% and 53% of non-naïve eyes (p < 0.001). A lack of anatomical response was rare in both groups, but more frequently in non-naïve eyes (12% vs. 4%, p = 0.003). Functionally and anatomically, naïve eyes showed most frequently an early and stable improvement (functionally: 77/175 44%; anatomically: 123/175 eyes, 70%). Most non-naïve eyes experienced no significant improvement functionally (97/242 eyes, 40%), despite a mostly early and stable improvement anatomical response pattern (102/242 eyes, 42%). Functional but not anatomical response patterns were influenced by baseline VA. Naïve and non-naïve eyes show different functional and anatomical response patterns to DEX implant. Functional non-responders are rare in naïve eyes, whereas anatomical non-response is unusual in both groups.

## Introduction

The number of people diagnosed with diabetes is expected to rise from 451 million cases in 2017 to 693 million cases in 2045^1^. Diabetic macular edema (DME) is a major health problem as it is a common cause of visual loss in working-aged patients^2^. With the advent of intravitreal vascular endothelial growth factor (VEGF) inhibitors, the outcomes of DME patients have significantly improved. Anti-VEGF agents are considered as first-line therapy for DME^3^. Randomized clinical trials (RCTs) and real-world studies have shown that about 30–40% of treatment-naïve DME patients respond well to anti-VEGF loading dose (3 monthly injections), but about the same proportion respond poorly or suboptimally (VA gain < 5 letters)^4–7^. Of these poor responders, half remain as poor responders even after 12 months of continuous treatment^4,7^. Alternate therapy with intravitreal corticosteroids, such as dexamethasone (DEX) intravitreal implant 0.7 mg (Ozurdex, Allergan, Inc., Irvine, CA, USA), have proven to be effective in DME^8–10^. Previous studies demonstrated a pendular effect profile of DEX implant, with a functional and anatomical peak response occurring after about 80 days^11^, a subsequent deterioration^8,11^ and a mean treatment effect duration of 4–5 months^12,13^.

Even though the proportion of initial functional response to anti-VEGF therapy is well known, data on the response rate to intravitreal therapy with DEX implant data is very limited. Hence, the primary purpose of this study was to evaluate the proportion of functional and anatomical responders and non-responders and to describe different response patterns to DEX implant in naïve and non-naïve DME cases. A grading or classification of the anatomical response to intravitreal therapy has not previously been reported, with most studies reporting a binary threshold to define anatomical response. Where reported, these thresholds have been inconsistent amongst different studies^14,15^. We therefore propose a grading classification of DME response to treatment, for both functional and anatomical parameters.

## Methods

This is an international multicenter cohort study comprising 11 study sites. Institutional review board (IRB) approval was obtained through the individual IRBs at the participating institutes for a retrospective consecutive chart review (see supplemental data). All approving IRB waived the need to obtain informed consent from study participants for this retrospective study. This research adhered to the tenets of the Declaration of Helsinki.

### Study participants

Medical records of consecutive patients from January 1st, 2011 to June 30th, 2018 with a diagnosis of DME were reviewed.

Inclusion criteria were^16^: (1) age of 18 years or older; (2) type 1 or 2 diabetes mellitus; (3) DME (both naïve and non-naïve) with study eye BCVA measuring 0.2 to 1.3 logarithm of the minimum angle of resolution (logMAR); DME were defined clinically and by retinal thickness of ≥ 300 µm in the central subfield; and intraretinal or subretinal fluid (SRF) seen on SD OCT; eyes with prior DME treatment had to present with persistent or recurrent DME ≥ 1 month after at least 3 monthly anti-VEGF injections, or ≥ 6 months after macular laser; (4) treatment with DEX implant, (5) minimum follow-up of 2 and 4 months after DEX implant. Only first treatments with DEX implant were considered for the study. For patients who received bilateral treatment with DEX, both eyes were included. Exclusion criteria were (1) concomitant ocular disease that causes macular edema (i.e., neovascular age-related macular degeneration or choroidal neovascularization due to other reasons, and recent intraocular surgery possibly causing postsurgical macular edema); (2) other conditions that compromises VA, except for the presence of cataract; and (3) previous treatment with intraocular corticosteroids; (4) last anti-VEGF injection < 30 days prior first DEX implant, (5) macular laser < 6 months prior first DEX implant.

### Data collection

The following data were collected: demographic data (i.e. age, sex); stage of diabetic retinopathy based on clinical examination (non-proliferative or proliferative); previous treatments (intravitreal therapy, macular laser); lens status at baseline; VA and CST at baseline, 2, and 4 months. The 2- and 4-month timepoints were chosen since previous studies showed a maximum DEX effect after 2 months and a mean treatment effect duration of 4 months in most cases^8,11–13^.

### Outcome measures

Main outcome measures were functional and anatomical response at month 2 and 4 in naïve and non-naïve eyes, as assessed by change in VA and CST from baseline. Functional response (FR) was graded as: very good FR (≥ + 10 letters gain from baseline), good FR (+ 5–9 letters gain from baseline), no FR (± 4 letters change from baseline), and significant vision loss (≤ -5 letters from baseline). Anatomical response (AR) was graded as: very good AR (> 40.0% reduction from baseline, or CST ≤ 300 µm), good AR (20.0–40.0% reduction from baseline), limited AR (10.0–19.9% reduction from baseline), poor AR (5.0–9.9% reduction from baseline), and no AR (< 5.0% reduction from baseline).

Treatment response patterns for functional response were graded as follows: early and stable improvement (month 2: very good FR or good FR; month 4: ± 4 letters change from month 2), early and progressive improvement (month 2: very good or good FR, month 4: ≥ 5 letters improvement from month 2), pendular response (month 2: very good or good FR, month 4: ≥ 5 letters loss from month 2), delayed improvement (month 2: no FR or severe vision loss, month 4: very good or good FR), persistent non-response (month 2 and month 4: no FR or severe vision loss).

Treatment response patterns for anatomical response were graded as follows: early and stable improvement (month 2: very good, good or limited AR; month 4: CST change ± 10% from month 2 and at least limited AR), early and progressive improvement (month 2: very good, good or limited AR, month 4: CST reduction > 10% from month 2), pendular response (month 2: very good, good or limited AR; month 4: CST increase > 10% from month 2), delayed improvement (month 2: poor or no AR, month 4: very good, good or limited AR) or persistent non-response (month 2 and month 4: poor or no AR, or limited AR at month 2 and poor or no AR at month 4 but CST increase < 10%).

### Statistical analysis

The demographic and clinical characteristics of our study cohort were evaluated using traditional descriptive methods. To control for the correlated nature of our data, we used a generalized estimating equations (GEE) procedure. Differences in outcome measures (VA/CST change at month 2 and 4) between naïve and non-naïve eyes were analyzed by logistic regression analysis, including baseline VA or CST respectively, and diabetic retinopathy status as imbalanced baseline characteristic. Statistical analysis was performed with SPSS Statistics 25 (IBM, Armonk, NY, USA).

## Results

In total 417 eyes from 388 subjects were included in the study. Demographic and baseline characteristics are shown in Table 1. Mean age was 64.7 ± 12.5 years. A total of 217 (52.9%) were phakic and 187 eyes (44.8%) had proliferative diabetic retinopathy (PDR). Most eyes (n = 242, 58%) had received previous treatments for DME, while 175 eyes (42%) were treatment naïve. Around half of the previously treated eyes (129/242, 53.3%) had focal/grid macular laser and 74.8% (181/242 eyes) had received anti-VEGF injections, with a mean 7.9 ± 7.8 injections per eye prior to first DEX implant (Table 1).

**Table 1:** Descriptive statistics: demographic data and baseline characteristics.

	All eyes (n = 417)	Naïve eyes (n = 175)	Non-naïve eyes (n = 242)	p-value*
Age, years, mean (SD)	64.7 (12.5)	66.3 (15.0)	63.6 (10.0)	0.060
Male gender, n (%)	245 (58.8)	103 (58.9)	142 (58.7)	0.972
Phakic eyes, n (%)	217/410 (52.9)	91 (52.0)	126/235 (53.6)	0.753
PDR, n (%)	187 (44.8)	96 (54.9)	91 (37.6)	0.001
Previous focal/grid laser, n (%)	129 (30.9)	0 (0)	129 (53.3)	–
Previous anti-VEGF injections, n (%)	181 (43.4)	0 (0)	181 (74.8)	–
No. previous anti-VEGF injections, mean (SD)	7.9 (7.8)	0 (0)	7.9 (7.8)	–

*p value between naïve and non-naïve eyes. Tested by univariable logistic regression analysis, generalized estimating equations model. PDR—Proliferative diabetic retinopathy, SD—Standard deviation, VEGF—Vascular endothelial growth factor.

### Functional response

Baseline VA at the time of first DEX implant was 0.63 ± 0.30 logMAR (naïve eyes: 0.68 ± 0.30 logMAR, non-naïve eyes: 0.60 ± 0.30 logMAR, p = 0.001, Table 2). Naïve eyes experienced a significantly greater VA gain at month 2 (+ 10.5 ± 12.3 letters vs. + 5.0 ± 10.0 letters, p < 0.001) and month 4 (+ 9.9 ± 13.8 letters vs. + 4.3 ± 11.8 letters, p = 0.006) compared to non-naïve eyes.

**Table 2:** Functional and anatomical outcomes.

	All eyes (n = 417)	Naïve eyes (n = 175)	Non-naïve eyes (n = 242)
VA prior DEX-I, logMAR, mean (SD)	0.63 (0.30)	0.68 (0.30)	0.60 (0.30)
Change in VA 2 months after DEX-I, letters, mean (SD)	+ 7.2 (11.4)	+ 10.5 (12.3)	+ 5.0 (10.0)
**Change in VA 2 months after DEX-I, n (%)**			
Very good FR (≥ + 10 letters)	178 (42.7)	98 (56.0)	80 (33.1)
Good FR (+ 5–9 letters)	89 (21.3)	45 (25.7)	44 (18.2)
No FR (± 4 letters)	101 (24.2)	22 (12.6)	79 (32.6)
Significant vision loss (≤ -5 letters)	49 (11.8)	10 (5.7)	39 (16.1)
Eyes with VA ≤ 0.3 logMAR 2 months after DEX-I, n (%)	140 (33.6)	64 (36.6)	76 (31.4)
Change in VA 4 months after DEX-I, letters, mean (SD)	+ 6.7 (13.0)	+ 9.9 (13.8)	+ 4.3 (11.8)
**Change in VA 4 months after DEX-I, n (%)**			
Very good FR (≥ + 10 letters)	166 (39.8)	99 (56.6)	67 (27.7)
Good FR (+ 5–9 letters)	94 (22.5)	48 (27.4)	46 (19.0)
No FR (± 4 letters)	92 (22.1)	14 (8.0)	78 (32.2)
Significant vision loss (≤ -5 letters)	65 (15.6)	14 (8.0)	51 (21.1)
Eyes with VA ≤ 0.3 logMAR 4 months after DEX-I, n (%)	135 (32.4)	62 (35.4)	73 (30.2)
CST prior DEX-I, µm, mean (SD)	534.5 (144.0)	547.3 (141.6)	525.2 (145.3)
Change in CST 2 months after DEX-I, µm, mean (SD)	− 217.0 (155.2)	− 243.0 (139.9)	− 198.3 (163.0)
**Change in CST 2 months after DEX-I, n (%)**			
Very good AR (> 40.0% reduction or CST ≤ 300 µm)	268 (64.3)	130 (74.3)	138 (57.0)
Good AR (20.0–40.0% reduction)	80 (19.2)	29 (16.6)	51 (21.1)
Limited AR (10.0–19.9% reduction)	33 (7.9)	10 (5.7)	23 (9.5)
Poor AR (5.0–9.9% reduction)	12 (2.9)	1 (0.6)	11 (4.5)
No AR (< 5.0% reduction)	24 (5.8)	5 (2.9)	19 (7.9)
Change in CST 4 months after DEX-I, µm, mean (SD)	− 166.3 (173.0)	− 223.3 (166.6)	− 124.9 (165.6)
**Change in CST 4 months after DEX-I, n (%)**			
Very good AR (> 40.0% reduction or CST ≤ 300 µm)	206 (49.4)	111 (63.4)	95 (39.3)
Good AR (20.0–40.0% reduction)	83 (19.9)	34 (19.4)	49 (20.2)
Limited AR (10.0–19.9% reduction)	39 (9.4)	10 (5.7)	29 (12.0)
Poor AR (5.0–9.9% reduction)	13 (3.1)	3 (1.7)	10 (4.1)
No AR (< 5.0% reduction)	76 (18.2)	17 (9.7)	59 (24.4)

AR—Anatomical response, CST—Central subfield thickness, DEX-I—Dexamethasone implant, FR—functional response, SD—Standard deviation, VA—Visual acuity.

### Grading of functional response

Grading of the functional response at month 2 and month 4 stratified for naïve and non-naïve eyes is shown in Table 2. In naïve eyes a total of 81.7% experienced a good or very good FR at month 2 and 84.0% after month 4. A significant VA loss (loss of ≥ 5 letters) was experienced in 5.7% (month 2) and 8.0% (month 4). Non-naïve eyes were less likely to experience a very good or good FR compared to naïve eyes (month 2: 51.3% vs. 81.7%, p < 0.001, Odds Ratio [OR]: 0.27, 95% confidence interval [CI]: 0.16–0.43; month 4: 46.7% vs. 84.0%, p < 0.001, OR: 0.19, 95% CI: 0.12–0.30). Grading of the functional response stratified for baseline VA is shown in Supplemental Table S1. Functional response after 2 and 4 months did differ significantly among the groups stratified for baseline VA (p ≤ 0.001 for month 2 and 4 respectively), with a higher likelihood of very good FR in eyes with worse baseline VA (Supplemental table S1).

### Functional response over time

**Figure 1:**
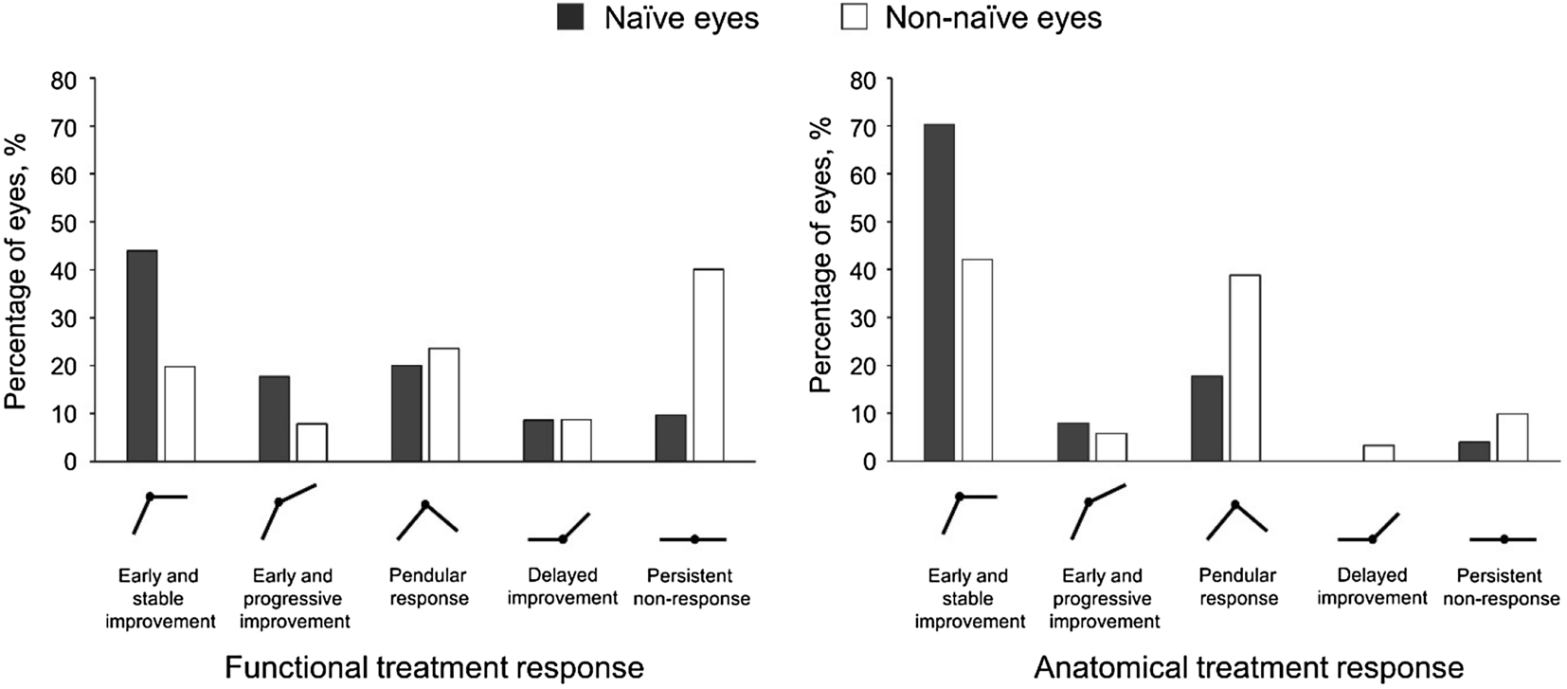
Functional and anatomical treatment response for naïve (black bars) and non-naïve eyes (white bars). Treatment response patterns for functional response (FR) were graded as follows: early and stable improvement (month 2: very good FR or good FR; month 4: ± 4 letters change from month 2), early and progressive improvement (month 2: very good or good FR, month 4: ≥ 5 letters improvement from month 2), pendular response (month 2: very good or good FR, month 4: ≥ 5 letters loss from month 2), delayed improvement (month 2: no FR or severe vision loss, month 4: very good or good FR), persistent non-response (month 2 and month 4: no FR or severe vision loss). Treatment response patterns for anatomical response (AR) were graded as follows: early and stable improvement (month 2: very good, good or limited AR; month 4: CST change ± 10% from month 2 and at least limited AR), early and progressive improvement (month 2: very good, good or limited AR, month 4: CST reduction > 10% from month 2), pendular response (month 2: very good, good or limited AR; month 4: CST increase > 10% from month 2), delayed improvement (month 2: poor or no AR, month 4: very good, good or limited AR) or persistent non-response (month 2 and month 4: poor or no AR, or limited AR at month 2 and poor or no AR at month 4 but CST increase < 10%).

In naïve eyes, a significant VA gain (≥ 5 letters from baseline) was maintained until month 4 in 95% of eyes with very good FR and in 87% of eyes with good FR at month 2 (Table 3). Naïve eyes with no FR at month 2, experienced a delayed response in 64% (45.5% good FR and 18.2% very good FR at month 4). An early and stable improvement response pattern was the most frequent pattern in naïve eyes (77/175, 44%, Fig. 1).

**Table 3:** Functional response at 4 months stratified for VA response after 2 months.

VA response 2 months after DEX-I	VA response 4 months after DEX-I (VA change from baseline), n (%)			
	Very good (≥ + 10 letters)	Good (+ 5–9 letters)	No change (± 4 letters)	Significant loss (≤ -5 letters)
**Naïve eyes, n = 175**				
Very good FR (≥ + 10 letters), n = 98	80 (81.6)	13 (13.3)	2 (2.0)	3 (3.1)
Good FR (+ 5–9 letters), n = 45	15 (33.3)	24 (53.3)	2 (4.4)	4 (8.9)
No FR (± 4 letters), n = 22	4 (18.2)	10 (45.5)	7 (31.8)	1 (4.5)
Significant VA loss (≤ -5 letters), n = 10	0 (0)	1 (10.0)	3 (30.0)	6 (60.0)
**Non-naïve eyes, n = 242**				
Very good FR (≥ + 10 letters), n = 80	52 (65.0)	16 (20.0)	8 (10.0)	4 (5.0)
Good FR (+ 5–9 letters), n = 44	9 (20.5)	15 (34.1)	11 (25.0)	9 (20.5)
No FR (± 4 letters), n = 79	5 (6.3)	8 (10.1)	50 (63.3)	16 (20.3)
Significant loss (≤ -5 letters), n = 39	1 (2.6)	7 (17.9)	9 (23.1)	22 (56.4)

DEX-I—Dexamethasone implant, FR—Functional response, VA—Visual acuity.

Non-naïve eyes were significant less likely to maintain VA gain throughout month 4 compared to naïve eyes. Non-naïve eyes with good FR at month 2 maintained VA gain (very good or good FR) in 55% of eyes (vs. 86% in naïve eyes, p = 0.009, OR: 0.22, 95% CI: 0.07–0.69). Non-naïve eyes with no FR at month 2 experienced a delayed functional response (very good or good at month 4) in 16% of eyes (vs. 64% in naïve eyes, p < 0.001, OR: 0.11, 95% CI: 0.04–0.31). The most frequent functional response pattern in non-naïve eyes was the persistent non-response pattern (97/242 eyes, 40%, Fig. 1). However, 60% of the eyes did gained vision in some point during follow-up (Fig. 1).

Functional response pattern stratified for baseline VA is shown in Supplemental Fig. 1. Eyes with good baseline VA (≤ 0.3 logMAR) were more likely to show a persistent non-response pattern compared to eyes with worse baseline VA (p = 0.003, OR: 1.66, 95% CI: 1.20–2.32). Eyes with worse baseline VA showed a pendicular response pattern more frequently than eyes with better baseline VA (p = 0.006, OR: 1.68, 95% CI: 1.16–2.44).

### Anatomical response

Baseline CST was 534.5 ± 144 µm with no difference between naïve (547.3 ± 14.6 µm) and non-naïve eyes (525.2 ± 145.3 µm, p = 0.134). CST decreased significantly in naïve and non-naïve eyes at month 2 (both groups: p < 0.001) and month 4 (both groups: p < 0.001; Table 2).

### Grading of anatomical response

A very good AR was seen in 74% of naïve eyes and in 57% of non-naïve eyes at month 2 (naïve vs. non-naïve: p = 0.004, OR: 1.95, 95% CI: 1.23–3.08) and at month 4 in 63% and 39% respectively (naïve vs. non-naïve eyes: p < 0.001, OR: 2.38, 95% CI: 1.56–3.64). A poor or no AR were seen at month 2 in 4% of naïve eyes and in 12% of non-naïve eyes (naïve vs. non-naïve eyes: p = 0.004, OR: 0.26, 95% CI: 0.10–0.65) and at month 4 in 11% and 29% respectively (naïve vs. non-naïve eyes: p = 0.001, OR: 0.37, 95% CI: 0.21–0.65). Grading of the anatomical response stratified for baseline VA is shown in Supplemental Table S1. Anatomical response after 2 and 4 months did not differ significantly among the groups (month 2: p = 0.167, month 4: p = 0.111).

### Anatomical response over time

A significant anatomical response (very good or good AR) at month 4 was maintained in 93% of naïve eyes and in 75% of non-naïve eyes with initial very good AR (naïve vs. non-naïve: p < 0.001, OR: 2.96; 95% CI: 1.67–5.24). An improvement by ≥ 1 grade after month 2 was seen in 21% of naïve eyes with good AR and in 30% with limited AR and was not seen in naïve eyes with poor or no AR at month 2 (Table 4). However, this delayed response was more likely in non-naïve eyes occurring in 36% with initial poor AR and in 42% with initial no AR. Conversely, significant DME recurrence (poor or no AR) at month 4 was more likely in non-naïve eyes and was observed in 17% of eyes with initial very good AR (vs. 5% in naïve eyes, p = 0.023, OR: 3.18, 95% CI: 1.17–8.66), in 29% of eyes with good AR (vs. 17% in naïve eyes, p = 0.264) and in 52% of eyes with initial limited AR (vs. 20% in naïve eyes, p = 0.066).

**Table 4:** Anatomical response at 4 months stratified for CST response after 2 months.

CST response 2 months after DEX-I (CST reduction from baseline)	CST response 4 months after DEX-I (CST reduction from baseline), n (%)				
	Very good (> 40.0%*)	Good (20.0–40.0%)	Limited (10.0–19.9%)	Poor (5.0–9.9%)	None (< 5.0%)
**Naïve eyes, n = 175**					
Very good (> 40.0%*), n = 130	104 (80.0)	17 (13.1)	2 (1.5)	1 (0.8)	6 (4.6)
Good (20.0–40.0%), n = 29	6 (20.7)	15 (51.7)	3 (10.3)	2 (6.9)	3 (10.3)
Limited (10.0–19.9%), n = 10	1 (10.0)	2 (20.0)	5 (50.0)	0 (0)	2 (20.0)
Poor (5.0–9.9%), n = 1	0 (0)	0 (0)	0 (0)	0 (0)	1 (100)
None (< 5.0%), n = 5	0 (0)	0 (0)	0 (0)	0 (0)	5 (100)
**Non-naïve eyes, n = 242**					
Very good (> 40.0%*), n = 138	77 (55.8)	26 (18.8)	12 (8.7)	3 (2.2)	20 (14.5)
Good (20.0–40.0%), n = 51	10 (19.6)	18 (35.3)	8 (15.7)	2 (3.9)	13 (25.5)
Limited (10.0–19.9%), n = 23	5 (21.7)	4 (17.4)	2 (8.7)	1 (4.3)	11 (47.8)
Poor (5.0–9.9%), n = 11	0 (0)	0 (0)	4 (36.4)	3 (27.3)	4 (36.4)
None (< 5.0%), n = 19	3 (15.8)	1 (5.3)	3 (15.8)	1 (5.3)	11 (57.9)

CST—Central subfield thickness. *or CST ≤ 300 µm.

Most naïve eyes showed an early and stable improvement response pattern (123/175 eyes, 70%). In non-naïve eyes an early and stable improvement response (102/242 eyes, 42%) and a pendular response (94/242 eyes, 39%) were the most frequent observed anatomical response pattern (Fig. 1). No significant difference in anatomical response patterns existed among the different baseline VA groups (p = 0.233, Supplemental Fig. 1).

## Discussion

This collaborative, multicenter, real-world study describes the functional and anatomical response to the first DEX implant in naïve and non-naïve DME eyes and provides detailed data on response patterns during the first 4 months. We also propose a new grading of treatment response in DME.

We showed that the proportion of eyes with very good functional response (VA gain ≥ 10 letters) was 56% and 57% at 2 and 4 months in naïve eyes; and 33% and 28% in non-naïve eyes respectively. The proportion of functional non-responders (VA gain < 5 letters) was 18% and 16% in naïve eyes (month 2 and 4), and 49% and 53% respectively in non-naïve eyes. While most studies focus on functional response, our study also provides a detailed analysis of the anatomical response. In our cohort most patients had a favorable anatomical response (very good or good AR: 91% for naïve, 78% for non-naïve) at 2 months while anatomical non-responders (CST reduction ≤ 10%) were rare in both groups (naïve eyes: 4%, non-naïve eyes: 12%). Most naïve DME eyes showed an early and stable functional and anatomical response to initial DEX implant therapy.

Many RCTs and real-life studies have described the proportion of functional responders and non-responders to anti-VEGF therapy, revealing that the proportion of very good functional responders (VA gain ≥ 10 letters) varies from 33 to 49%, and the proportion of non-responders (VA gain < 5 letters) ranges from 26 to 40% after 3 monthly anti-VEGF injections^4–7^. However, data on the proportion of responders and non-responders to an initial DEX implant have been rare so far. While most previous studies on the response rate to anti-VEGF therapy included naïve eyes, most studies on DEX implant reported on non-naïve eyes only or mixed cohorts^8,11,12^. Belloq et al. described in a 6-month follow-up study of a cohort of 37 mostly naïve eyes (73% naïve eyes), that 84% achieved a VA gain of ≥ 5 letters while 16% of those eyes were functional non-responders^12^, confirming our results of a low proportion of functional non-responders to DEX implant among naïve eyes. Our data suggests that for naïve eyes, proportion of functional non-responders is less when compared to anti-VEGF loading dose. However, a head-to-head prospective randomized controlled trial in naïve eyes is necessary. The BEVORDEX trial^17^ was the first and so far, only prospective trial, which directly compared bevacizumab and DEX implant in DME. However, this trial included eyes after at least 3 months of at least 1 session of macular laser treatment or those for which macular laser found to be unhelpful^18^, thus being reflective of non-naïve eyes. A post hoc analysis of the BEVORDEX trial^17^ revealed similar non-response rates after 3 months (17/35 eyes, 49%) as we reported in our study for non-naïve eyes after 2 and 4 months.

To our knowledge, our study is the largest cohort of naïve eyes that demonstrates that the proportion of responders and non-responders to initial DEX implant differs profoundly between naïve and non-naïve eyes. It has been shown before that the outcome of naïve eyes to DEX implant is significantly better compared to non-naïve eyes^9,10,19^. Hence, it needs to be emphasized that when comparing the response rate to DEX implant between studies, proportion of naïve eyes in those studies needs to be considered. Furthermore, in our study naïve eyes fared significantly better in terms of CST changes with a higher proportion of eyes with very good anatomical response compared to non-naïve eyes, confirming our previous report^9^. Despite the low presence of anatomical non-responders, a very large number of eyes in the non-naïve group showed a persistent functional non-response. Other factors such as irreversible damage from chronic macular edema or decreased macular perfusion may prevent these eyes from having an improvement in vision despite a good anatomical response.

Our study provides detailed data on treatment effect variations during the first 4 months of treatment. While functional response was stable in most naïve eyes, with only 20% of eyes experiencing a significant VA worsening (≥ 5 letters) after 2 months, functional response worsening occurred in 39% of non-naïve eyes. Previous studies described this pendular response pattern being typical for DEX implant^8,11^. However, our study analyzed different treatment patterns in naïve and non-naïve eyes, revealing that for naïve eyes an early and stable improvement was the most frequently observed response pattern functionally and anatomically. In non-naïve eyes a persistent non-response was the most common functional response pattern despite 60% having improved some vision during follow-up. Regarding anatomical response in non-naïve eyes both patterns, early and stable improvement and pendular response, were equally likely. This relatively frequent pendular anatomical response pattern in non-naïve eyes (39%) suggests that more frequent DEX injections may be needed than in naïve eyes (18%). Hence, non-naïve eyes might require tighter follow-up intervals than naïve eyes. Interestingly, even when naïve eyes present no VA change at 2 months, chances of a VA gain ≥ 5 letters at 4 months were 64% (compared to 16% in non-naïve eyes). Our study only evaluated the response to the first DEX implant, since early studies suggested that treatment interval tend to increase, when DEX implant is applied repeatedly with an increased response variability^10,13^.

Besides treatment-naivity also baseline VA influenced functional response rate to DEX implant. The potential of eyes with baseline VA ≤ 0.3 logMAR to achieve a significant VA gain was significantly less than eyes with worse baseline VA. Baseline VA has been shown as a confounder for treatment outcome before, as a ceiling effect may limit the number of letters gained for patients with a good initial VA^16,20^.

Limitations of this study include its retrospective nature and shortcomings of a real-world setting. We did not evaluate the influence of baseline characteristics (except baseline VA), such as HbA1c level or existing comorbidities on functional and anatomical response and response pattern. Furthermore, we did not analyze whether baseline OCT features are associated with different treatment response patterns, which needs to be addressed in future studies. Baseline characteristics between naïve and non-naïve eyes were not matched in terms of PDR presence, which has been accounted for in the statistical analysis. Furthermore, chronicity of the edema, macular perfusion status and cataract grading were not evaluated. Used OCT devices varied between the study centers which might have influenced the results. And finally, our study was limited to a primary endpoint of 4 months.

In conclusion, this study revealed that the proportion of functional and anatomical non-responders to DEX implant are low in naïve eyes. An anatomical non-response is rarely seen in naïve and non-naïve eyes. DME recurrence at month 4 is more likely in non-naïve eyes, but unlikely in naïve eyes. Most naïve eyes show an early and stable anatomical and functional improvement to DEX implant, while non-naïve eyes had a higher risk for experiencing no functional benefit despite a significant anatomical improvement after the first DEX implant. Baseline VA influences the likelihood of significant VA gains. We also proposed a new comprehensive grading system for DME treatment response.

## Supplementary information

Supplementary Information.

## References

[1] Cho NH (2018). IDF Diabetes Atlas: global estimates of diabetes prevalence for 2017 and projections for 2045. Diabetes Res. Clin. Pract..

[2] Yau JW (2012). Global prevalence and major risk factors of diabetic retinopathy. Diabetes Care.

[3] Schmidt-Erfurth U (2017). Guidelines for the Management of Diabetic Macular Edema by the European Society of Retina Specialists (EURETINA). Ophthalmol. J. Int. d'ophtalmologie Int. J. Ophthalmol. Zeitschrift fur Augenheilkunde.

[4] Gonzalez VH (2016). Early and long-term responses to anti-vascular endothelial growth factor therapy in diabetic macular edema: analysis of protocol I data. Am. J. Ophthalmol..

[5] Bressler NM (2018). Early response to anti-vascular endothelial growth factor and two-year outcomes among eyes with diabetic macular edema in protocol T. Am. J. Ophthalmol..

[6] Santos AR (2018). Optical coherence tomography baseline predictors for initial best-corrected visual acuity response to intravitreal anti-vascular endothelial growth factor treatment in eyes with diabetic macular edema: the CHARTRES Study. Retina.

[7] Maggio E (2018). Anti-vascular endothelial growth factor treatment for diabetic macular edema in a real-world clinical setting. Am. J. Ophthalmol..

[8] Boyer DS (2014). Three-year, randomized, sham-controlled trial of dexamethasone intravitreal implant in patients with diabetic macular edema. Ophthalmology.

[9] Iglicki M (2018). Dexamethasone implant for diabetic macular edema in naive compared with refractory eyes: The International Retina Group Real-Life 24-Month Multicenter Study. The IRGREL-DEX Study. Retina.

[10] Malcles A (2017). Real-life study in diabetic macular edema treated with dexamethasone implant: the Reldex Study. Retina.

[11] Rosenblatt A (2020). A collaborative retrospective study on the efficacy and safety of intravitreal dexamethasone implant (Ozurdex) in patients with diabetic macular edema: the European DME Registry Study. Ophthalmology.

[12] Bellocq D (2018). The pattern of recurrence in diabetic macular edema treated by dexamethasone implant: the PREDIAMEX Study. Ophthalmol. Retina.

[13] Mehta H, Fraser-Bell S, Nguyen V, Lim LL, Gillies MC (2018). The interval between treatments of bevacizumab and dexamethasone implants for diabetic macular edema increased over time in the BEVORDEX Trial. Ophthalmol. Retina.

[14] Dugel PU (2019). Association between early anatomic response to anti-vascular endothelial growth factor therapy and long-term outcome in diabetic macular edema: an independent analysis of protocol I study data. Retina.

[15] Pieramici DJ, Wang PW, Ding B, Gune S (2016). Visual and anatomic outcomes in patients with diabetic macular edema with limited initial anatomic response to Ranibizumab in RIDE and RISE. Ophthalmology.

[16] Zur D (2017). Optical coherence tomography biomarkers as functional outcome predictors in diabetic macular edema treated with dexamethasone implant. Ophthalmology.

[17] Mehta H, Fraser-Bell S, Nguyen V, Lim LL, Gillies MC (2018). Short-term vision gains at 12 weeks correlate with long-term vision gains at 2 years: results from the BEVORDEX randomised clinical trial of bevacizumab versus dexamethasone implants for diabetic macular oedema. Br. J. Ophthalmol..

[18] Gillies MC (2014). A randomized clinical trial of intravitreal bevacizumab versus intravitreal dexamethasone for diabetic macular edema: the BEVORDEX study. Ophthalmology.

[19] Escobar-Barranco JJ, Pina-Marin B, Fernandez-Bonet M (2015). Dexamethasone implants in patients with naive or refractory diffuse diabetic macular edema. Ophthalmol. J. Int. d'ophtalmologie Int. J. Ophthalmol. Zeitschrift fur Augenheilkunde.

[20] Sophie R, Lu N, Campochiaro PA (2015). Predictors of functional and anatomic outcomes in patients with diabetic macular edema treated with ranibizumab. Ophthalmology.

