# Supplementary material for: GRAding of functional and anatomical response to DExamethasone implant in patients with Diabetic Macular Edema: GRADE-DME Study
Source: Sci Rep. 2021 Feb 26;11:4738. doi: 10.1038/s41598-020-79288-w (PMC7910444; doi:10.1038/s41598-020-79288-w)
Supplement: Supplementary file 1 — Supplementary Information. [file 41598_2020_79288_MOESM1_ESM.docx]

**Supplemental Data**

**GRAding of functional and anatomical response to DExamethasone implant in patients with Diabetic Macular Edema - GRADE-DME Study**

Patricio J. Rodríguez-Valdés^1*^, Matus Rehak^2*^, Dinah Zur^3,4^, Anna Sala-Puigdollers^5^, Samantha Fraser-Bell^6^, Marco Lupidi^7^, Jay Chhablani^8,9^, Zafer Cebeci^10^, Inês Laíns^11,12^, Voraporn Chaikitmongkol^13^, Adrian T. Fung^6, 14, 15^, Mali Okada^16^, Jan Darius Unterlauft^2^, Lital Smadar^3,4^, Anat Loewenstein^3,4,17^, Matias Iglicki^18**^, Catharina Busch^2**^

**For the International Retina Group**

^1^ Instituto de Oftalmología y Ciencias Visuales, Escuela de Medicina, Tecnológico de Monterrey, Monterrey, Mexico

^2^ Department of Ophthalmology, University Hospital Leipzig, Germany

^3^ Division of Ophthalmology, Tel Aviv Sourasky Medical Center, Tel Aviv, Israel

^4^ Sackler Faculty of Medicine, Tel Aviv University, Tel Aviv, Israel

^5^ Institut Clínic d'Oftalmología (ICOF), Hospital Clinic de Barcelona, Barcelona, Spain

^6^ Department of Ophthalmology, Sydney University, Sydney, Australia

^7^ Department of Surgical and Biomedical Sciences, Section of Ophthalmology, University of Perugia, Italy

^8^ University of Pittsburgh, UPMC Eye Center, Pittsburgh, US

^9^ L.V. Prasad Eye Institute, Banjara Hills, Hyderabad, India

^10^ Istanbul University, Istanbul Faculty of Medicine, Department of Ophthalmology, Istanbul, Turkey

^11^ Massachusetts Eye and Ear, Harvard Medical School, Boston, US

^12^ Association for Innovation and Biomedical Research on Light, Coimbra, Portugal

^13^ Retina Division, Department of Ophthalmology, Faculty of Medicine, Chiang Mai University, Chiang Mai, Thailand

^14^ Westmead and Central Clinical Schools, Discipline of Ophthalmology and Eye Health, The University of Sydney, Sydney, Australia

^15^ Faculty of Medicine, Health and Human Sciences, Macquarie University Hospital, Sydney, Australia

^16^ Royal Victorian Eye and Ear Hospital, Melbourne, Victoria, Australia

^17^ Incumbent, Sydney A. Fox chair in Ophthalmology, Tel Aviv University, Tel Aviv, Israel

^18^ Private Retina Service, University of Buenos Aires, Buenos Aires, Argentina

*Equal contribution

**Equal contribution

**SD1. Participating study centers and approving institutional review boards.**

1. Instituto de Oftalmología y Ciencias Visuales, Escuela de Medicina, Tecnológico de Monterrey, Monterrey, Mexico.
2. Department of Ophthalmology, University Hospital Leipzig, Germany.
3. Division of Ophthalmology, Tel Aviv Sourasky Medical Center, Tel Aviv, Israel.
4. Institut Clínic d'Oftalmología (ICOF), Hospital Clinic de Barcelona, Barcelona, Spain.
5. Department of Ophthalmology, Sydney University, Sydney, Australia.
6. Department of Surgical and Biomedical Sciences, Section of Ophthalmology, University of Perugia, Italy.
7. L.V. Prasad Eye Institute, Banjara Hills, Hyderabad, India.
8. Istanbul University, Istanbul Faculty of Medicine, Department of Ophthalmology, Istanbul, Turkey.
9. Retina Division, Department of Ophthalmology, Faculty of Medicine, Chiang Mai University, Chiang Mai, Thailand.
10. Association for Innovation and Biomedical Research on Light, Coimbra, Portugal.
11. Private Retina Service, University of Buenos Aires, Buenos Aires, Argentina.

**SD2 Supplemental Table 1. Functional and anatomical outcomes, stratified for baseline VA.**

|  | **Baseline VA ≤0.30 logMAR**  **(n=70 eyes)** | **Baseline VA 0.32 – 0.70 logMAR**  **(n=223 eyes)** | **Baseline VA >0.70 logMAR**  **(n=124 eyes)** |
| --- | --- | --- | --- |
| VA prior DEX-I, logMAR, mean (SD) | 0.27 (0.05) | 0.55 (0.11) | 0.99 (0.25) |
| Change in VA 2 months after DEX-I, letters, mean (SD) | 2.7 (6.6) | 5.4 (9.6) | 13.3 (13.8) |
| Change in VA 2 months after DEX-I, n (%)  Very good FR (≥ +10 letters)  Good FR (+5-9 letters)  No FR (± 4 letters)  Significant vision loss (≤ -5 letters) | 15 (21.4)  22 (31.4)  21 (30.0)  12 (17.1) | 89 (39.9)  50 (22.4)  52 (23.3)  32 (14.3) | 74 (59.7)  17 (13.7)  28 (22.6)  5 (4.0) |
| Eyes with VA ≤ 0.3 logMAR 2 months after DEX-I, n (%) | 60 (85.7) | 69 (30.9) | 11 (8.9) |
| Change in VA 4 months after DEX-I, letters, mean (SD) | 2.5 (7.7) | 5.0 (10.4) | 11.9 (17.2) |
| Change in VA 4 months after DEX-I, n (%)  Very good FR (≥ +10 letters)  Good FR (+5-9 letters)  No FR (± 4 letters)  Significant vision loss (≤ -5 letters) | 16 (22.9)  18 (25.7)  21 (30.0)  15 (21.4) | 87 (39.0)  55 (24.7)  40 (17.9)  41 (18.4) | 63 (50.8)  21 (16.9)  31 (25.0)  9 (7.3) |
| Eyes with VA ≤ 0.3 logMAR 2 months after DEX-I, n (%) | 57 (81.4) | 67 (30.0) | 11 (8.9) |
| CST prior DEX-I, µm, mean (SD) | 466.0 (101.9) | 520.8 (133.9) | 597.8 (157.8) |
| Change in CST 2 months after DEX-I, µm, mean (SD) | -150.4 (115.8) | -205.5 (140.4) | -275.5 (179.1) |
| Change in CST 2 months after DEX-I, n (%)  Very good AR (> 40.0% reduction or CST ≤ 300µm)  Good AR (20.0-40.0% reduction)  Limited AR (10.0-19.9% reduction)  Poor AR (5.0-9.9% reduction)  No AR (< 5.0% reduction) | 41 (58.6)  15 (21.4)  6 (8.6)  3 (4.3)  5 (7.1) | 139 (62.3)  45 (20.2)  20 (9.0)  9 (4.0)  10 (4.5) | 88 (71.0)  20 (16.1)  7 (5.6)  0 (0)  9 (7.3) |
| Change in CST 4 months after DEX-I, µm, mean (SD) | -95.9 (117.4) | -161.4 (168.0) | -214.5 (192.6) |
| Change in CST 4 months after DEX-I, n (%)  Very good AR (> 40.0% reduction or CST ≤ 300µm)  Good AR (20.0-40.0% reduction)  Limited AR (10.0-19.9% reduction)  Poor AR (5.0-9.9% reduction)  No AR (< 5.0% reduction) | 28 (40.0)  11 (15.7)  12 (17.1)  5 (7.1)  14 (20.0) | 112 (50.2)  44 (19.7)  20 (9.0)  7 (3.1)  40 (17.9) | 66 (53.2)  28 (22.6)  7 (5.6)  1 (0.8)  22 (17.7) |

AR – Anatomical response, CST – Central subfield thickness, DEX-I – Dexamethasone implant, FR – functional response, SD – Standard deviation, VA – Visual acuity.


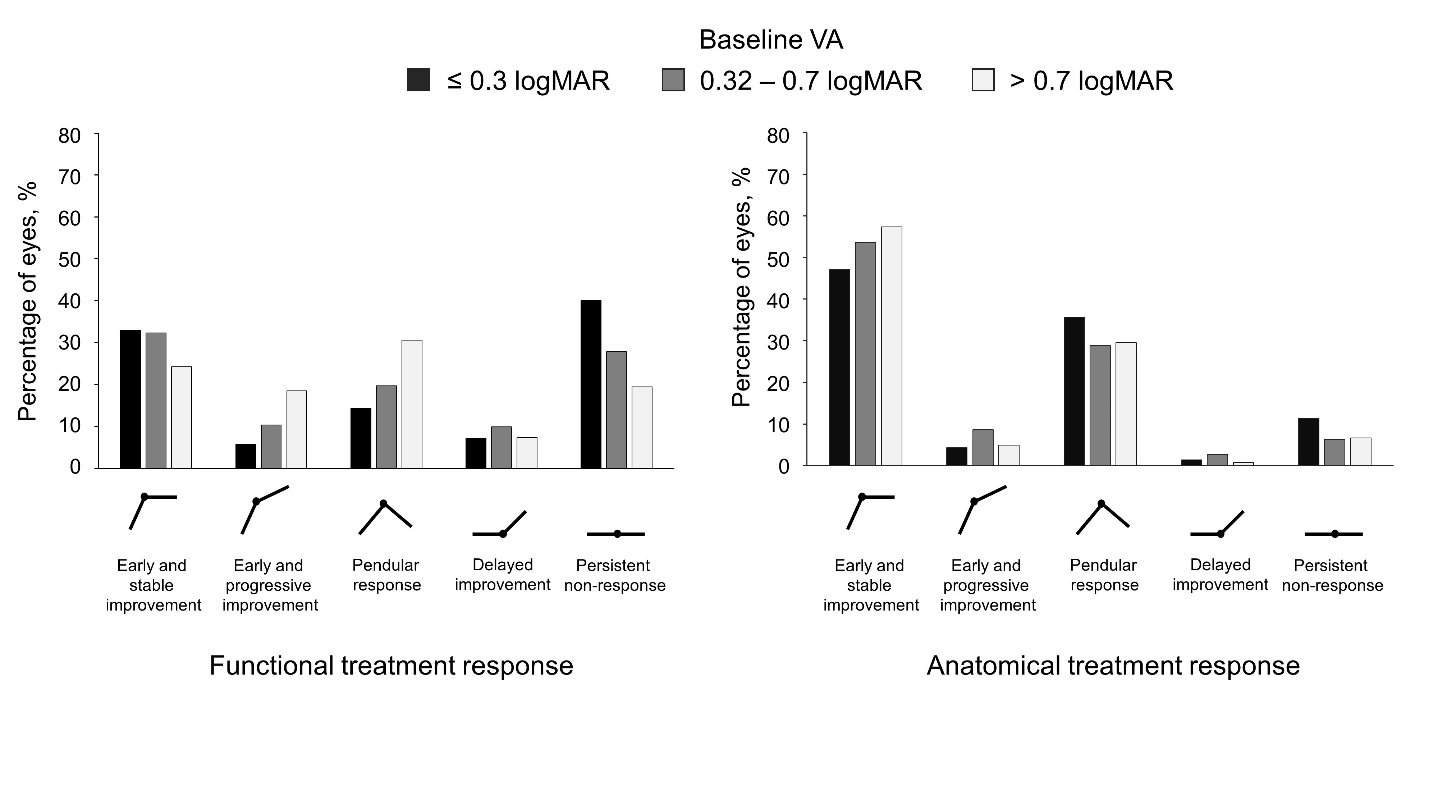


**SD3 Supplemental Figure 1.** Functional and anatomical treatment response for eyes with baseline visual acuity of ≤ 0.3 logMAR (black bars), 0.32 – 0.7 logMAR (grey bars) and > 0.7 logMAR (white bars). Treatment response patterns for functional response (FR) were graded as follows: early and stable improvement (month 2: very good FR or good FR; month 4: ± 4 letters change from month 2), early and progressive improvement (month 2: very good or good FR, month 4: ≥ 5 letters improvement from month 2), pendular response (month 2: very good or good FR, month 4: ≥ 5 letters loss from month 2), delayed improvement (month 2: no FR or severe vision loss, month 4: very good or good FR), persistent non-response (month 2 and month 4: no FR or severe vision loss). Treatment response patterns for anatomical response (AR) were graded as follows: early and stable improvement (month 2: very good, good or limited AR; month 4: CST change ± 10% from month 2 and at least limited AR), early and progressive improvement (month 2: very good, good or limited AR, month 4: CST reduction > 10% from month 2), pendular response (month 2: very good, good or limited AR; month 4: CST increase > 10% from month 2), delayed improvement (month 2: poor or no AR, month 4: very good, good or limited AR) or persistent non-response (month 2 and month 4: poor or no AR, or limited AR at month 2 and poor or no AR at month 4 but CST increase < 10%).
